# GEANT4 Simulation for Radioactive Particle Tracking (RPT) Technique

**DOI:** 10.3390/s22031223

**Published:** 2022-02-05

**Authors:** Ahmed A. Alghamdi, Thaar M. Aljuwaya, Abdullah S. Alomari, Muthanna H. Al-Dahhan

**Affiliations:** 1 Nuclear Science Research Institute, King Abdulaziz City for Science and Technology (KACST), Riyadh 11442, Saudi Arabia; ahmedg@kacst.edu.sa (A.A.A.); asalomari@kacst.edu.sa (A.S.A.); 2 Nuclear Engineering Department, Missouri University of Science and Technology (Missouri S&T), Rolla, MO 65409, USA; aldahhanm@mst.edu; 3 Chemical and Biochemical Engineering Department, Missouri University of Science and Technology (Missouri S&T), Rolla, MO 65409, USA

**Keywords:** localization and object tracking, radioactive particle tracking (RPT), radiotracer, Monte Carlo simulation, radiation detector

## Abstract

In the past two decades, the radioactive particle tracking (RPT) measurement technique has been proven to visualize flow fields of most multiphase flow systems of industrial interest. The accuracy of RPT, and hence the data obtained, depend largely on the calibration process, which stands here as a basis for two subsequent processes: tracking and reconstruction. However, limitations in the RPT calibration process can be found in different experimental constrains and in assumptions made in the classical Monte Carlo approach used to simulate number of counts received by the detectors. Therefore, in this work, we applied a GEANT4-based Monte Carlo code to simulate the RPT calibration process for an investigated multiphase flow system (i.e., gas–liquid bubble column). The GEANT4 code was performed to simulate the number of counts received by 28 scintillation detectors for 931 known tracer positions while capturing all the types of photon interaction and overcoming solids’ angle limitations in classical approaches. The results of the simulation were validated against experimental data obtained using an automated RPT calibration device. The results showed a good agreement between the simulated and experimental counts, where the maximum absolute average relative deviation detected was about 5%. The GEANT4 model typically predicted the number of counts received by all the detectors; however, it over-estimated the counts when the number of primary events applied in the model was not the optimal.

## 1. Introduction

Radioisotope-based measurement techniques have found many applications in different sectors of industry as well as in research and development. The radioisotopes measurement techniques have contributed to a wide range of process industries such as monitoring, inspection, process troubleshooting, optimization, control, and many other purposes [1]. An example of that can be found in petrochemical industry, where such techniques are very effective in monitoring and mapping the most multiphase flow systems of industrial interest. In multiphase flow systems, two-phase, or three-phase flows (or above) take place interacting with each other in different flow regimes and forms. It emphasizes the fact that characterization of multiphase flow systems is a challenging process since these systems include a large volume of the dispersed phase, opaque, and operate at the churn-turbulent flow regime [2]. Among the different radioisotope-based measurement techniques reported in the literature, the radioactive particle tracking (RPT) technique has a proven capability to visualize flow fields of multiphase flow systems. The RPT technique is a Lagrangian measurement technique, used to track the movement of a single radioactive tracer by means of detectors to obtain the velocity field of a multiphase flow system such as gas–liquid bubble column [3], gas–solid spouted beds [4,5], gas–solid riser [6,7,8], and gas–solid fluidized beds [9,10]. It has been proven through research studies and practical experimentation that the RPT is the only non-invasive technique that can provide a full description of a 3D flow field of highly opaque multiphase flow systems [2]. The RPT is a promising tool for laboratory and pilot plant research and for on-site industrial application in advancing performance, design, and scale-up of the above systems. In addition, data obtained from the RPT technique are very valuable as benchmarking data for assessing and validating computational fluid dynamics (CFD) models and their closures. Given the potential capabilities of the RPT technique, the implementation of this technique in the abovementioned systems is not trivial, nor is the optimal design [11]. With that perspective mentioned, research and development is required for proper implementation of the RPT technique, and this is the prime motivation of the present work.

In the RPT, a single tracer particle embedded with a selected unstable radioactive isotope, a gamma-ray emitter, is tracked using an array of detectors to visualize the flow filed of a multiphase flow system. Among various types of radioisotopes, the most common used in the RPT are Cobalt-60, Gold-198, and Scandium-46. The tracer particle is to be proactively designed for manufacturing to dynamically match the phase (either liquid or solid phase) whose motion is to be tracked. For tracking particulate solids (e.g., [4,5,12]), a tracer particle is designed to match the physical properties of the solid particles of the system. A tracer particle is of the same size, density, and shape as the solid particles of the system is manufactured. For tracking liquids, (e.g., [13]), a neutrally buoyant tracer with a composite density similar to the density of the liquid phase of interest is manufactured. In addition, the size of the tracer particle for tracking liquid should be made as small as possible (i.e., 1 mm or below) to reduce the drag force. Following the preparation of the tracer particle, three consecutive processes should take place for the RPT experimentations:

1. Calibration process (counts and corresponding tracer positions are known);
2. Actual tracking process (counts are known while instantaneous positions of tracer are unknown);
3. Reconstruction process (reconstruction algorithm is used to identify instantaneous positions of tracer).

In the calibration process, the tracer particle is placed “static” at different known positions in the column (i.e., reactor vessel) of the investigated multiphase system while collecting counts of gamma-rays received by all the detectors. As a result, a distance count map relating the tracer-to-detector distance with the collected counts is produced for each one of the detectors. The distance count map produced out of the calibration is the foundation for successful implementation of the RPT technique, and hence the goodness of the results acquired. In the tracking experiment, the tracer particle is left inside the column of the investigated system to move freely while the detectors are collecting the gamma-rays emitted by the tracer at a selected time interval. As a result, counts are produced out from the tracking run for all the detectors, while instantaneous positions of the tracer particle are unknown, and hence a reconstruction process is needed. The tracking and the calibration experiments are both conducted at identical operating conditions of the investigated multiphase flow system. In the reconstruction process, a reconstruction algorithm is applied on the actual tracking data to reconstruct the instantaneous positions of the tracer with the aid of the calibration data. Different reconstruction algorithms have been reported in the literature for such a purpose as follows: Data Reduction Scheme [10,14,15], Monte Carlo-Based Search Method [16,17], Neural Network-Based Method [18], and Cross-Correlation-Based Search Method involving Semi-Empirical Model [19]. Finally, the output of the reconstruction process is the Lagrangian time-series data of the tracer (i.e., time differentiation of instantaneous position). Consequently, the obtained Lagrangian time-series data provide abundant information about the tracked phase flow fields such as the 3D mean velocity fields, shear stresses, turbulence parameters, and dispersion coefficients, along with other parameters that depict characteristics and flow regimes and of the tagged flow phase [1]. As mentioned previously, the RPT is not ready-made technique and, hence, needs customize implementation to suit the investigated multiphase flow system and to obtain reliable data. Having that said, it is clear from the above discussion that the calibration process is the key for successful implantation of the RPT. It has been reported that the accuracy of the RPT experimentations depends extremely on the calibration process [11]. Consequently, investigation and development are needed to improve the RPT calibration process.

In the literature, several studies have reported towards the improvement of the RPT technique/calibration process [11,20,21]. Theoretically, the number of photopeak counts C that would be detected by a detector with energy source activity A, and in counting time T with respect to the total detection efficiency (*ε*), is given by [22] as follows:

(1) $$C=\frac{T\upsilon A\phi \varepsilon }{1+\tau \upsilon A\phi \varepsilon }$$

where

(2) $$\varepsilon ={∯}_{\Omega }^{}\frac{\underline{r}.\underline{n}}{r^{3}}\underset{f_{a}}{\underbrace{exp\left(-\mu_{R}e_{R}-\mu_{w}e_{w}\right)}}\underset{f_{D}}{\underbrace{\left(1-\exp\left(-\mu_{D}d\right)\right)d\Sigma }}$$

A detailed description for each symbol in Equations (1) and (2) is presented in Abbreviation.

Hence, Equation (1) provides the mathematical description of the phenomenological relation between the counts detected by the detector and the location of the point source (i.e., tracer particle in the RPT case). However, it is still difficult to calculate the absolute detector efficiency in Equation (2), as well as computing Equation (1). This difficulty is resulted from two reasons. The first reason is that the solid angle subtended by the detector surface area for each source position in the calibration process, either axial or non-axial, is unknown. The second reason is that the penetration depth of photons through the detector crystal (i.e., the absorption probability of the photon incident on the detector crystal) is unknown. Consequently, it is then necessary to apply a numerical solution as a way to evaluate the two quintinites, and hence find the total detection efficiency [11,20,23,24]. The authors of [23] have laid out the foundation of the Monte Carlo application for the calculation of total intrinsic, peak-to-total ratio efficiencies of cylindrical NaI(Tl) detector for a monoenergetic gamma-ray source located at any arbitrary position in air. In the Monte Carlo approach, photon path histories are tracked on their journey from the point source, throughout the medium, to the surface of the detector. It has been demonstrated that detector efficiencies at any specified energy can be obtained using the Monte Carlo approach rather than the need for tedious laboratory experiments. However, the effect of intervening medium between the source and the detector was not included in the calculations of [23]. In the RPT, the intervening medium between the source and the detector includes the multiphase flow distributions of the reactor itself, the reactor column wall material, the detector crystal housing material, the photomultiplier tube of the detector, and the tracer particle composition materials. On the other hand, several studies have indicated that the aforementioned effect is only considerable in case the entire energy spectrum is simulated, which is not the situation in the RPT technique [25,26]. In the RPT, only the photo-peak region of the spectrum is taken into account in the calibration (as well in the tracking) process regardless of whether it is done experimentally or through modeling. Additionally, it has been reported that the inclusion of the effects of detector housing material is necessary to increase similarity between the experiments and Monte Carlo calculations [25]. Another limitation in the Monte Carlo approach of [23] is that only Compton and photoelectric interactions are considered in the calculations. This means that the selection of radioisotopes for the RPT technique should be limited for those with photon energies of 1.022 MeV or less. Additionally, it is to be mentioned that production of secondary charged particles (electrons) is neglected, which reflects the deposition of all energy at the detector. Subsequently, the authors of [20] adopted the Monte Carlo approach for the first time in the RPT framework to compute the absolute detector efficiencies in the presence of intervening medium, and hence generating the counting map of the RPT calibration process. However, there are still three parameters that need to be tuned experimentally before the implementation of the Monte Carlo model. These parameters are as follows: the dead-time of counting system, tracer radioactivity strength, and linear attenuation coefficient of the reactor medium. The model was computed for eight NaI detectors and 19,200 tracer positions in a three-phase fluidized bed reactor. The authors of [20] validated the model experientially for 150 positions by placing the tracer manually inside the fluidized bed while collecting counts by the detectors using a probe calibration device. The authors of [11] applied the same approach to assess various parameters in the RPT experimentation such as the selected radioisotope, source strength, crystal size, crystal shape, crystal material, and detector elevation. However, a drawback in the calculations of [11,20] can be found in the estimation of the photo-peak efficiency, which was estimated from the ratio of the photo peak to total efficiencies, which also was assumed constant. In addition, the impact on the phase’s distributions due to alteration in the applied operating conditions was taken as constant [20].

Considering this state-of-the-art technique, Monte Carlo calculations found in the literature for the RPT calibration process are rather simplified by assumptions/estimations. Therefore, more accurate models are needed for sufficiently accurate RPT calibration process, and hence, successful RPT experimentation and reliable data can be obtained. Such models should be able to capture all the types of photon interaction with matter and should not be limited to solids angle determined by the detectors. This possibly can be achieved through common Monte Carlo based codes available in the literature for photon transport simulations such as MCNP [27,28] and GEANT4 [29]. To the best of the authors’ knowledge, the only studies applying such codes for the RPT calibration process are so far reported by [21,30,31]. The authors of [21,32] applied the so-called Monte Carlo N-Particle code version 5 (MCNP5) to simulate the RPT calibration process and proposed a new algorithm for the reconstruction process. Recently, substantial progress has been made in developing a user-friendly software called “GIPPE-RPT” for the RPT calibration process [30]. The GIPPE-RPT software is mainly embedded with a GEANT4-based Monte Carlo model coupled with a graphical user interface and used for estimating the photon counts received by the detectors. The software can be used for different RPT cases regarding geometry and materials of the investigated multiphase flow system, tracer particle type and activity, detector number and type, and detectors’ configuration around the investigated system. The GIPPE-RPT software also is integrated by a computational fluid dynamics (CFD) solver to estimate, and transfer to the GEANT4, the phase holdup distribution of the investigated multiphase flow system. The authors of [31] also applied the GIPPE-RPT software with the help of the transfer learning (TL) approach to examine the re-using of historical calibration data when the operation conditions of the system have been changed. They concluded that the TL approach can be used to exploit historical RPT calibration data collected under various conditions when training an RPT model under a new condition. In particular, the TL approach could be very useful for challenging RPT scenarios where it is difficult or sometimes impossible to collect sufficient RPT calibration data. Example of that can be found in high-pressure or high-temperature multiphase flow systems or at the presence of dense internals inside the system. Additionally, it was concluded that the inclusion of historical data by means of the TL approach, when sufficient RPT calibration data for a new condition is available, showed less reconstruction accuracy, and hence is not recommended [31]. However, despite major progress made using these Monte Carlo-based models [21,30,31], the experimental validation for such models is a must for a more reliable RPT calibration process. The main process in the RPT technique is the actual tracking, which is an absolute experimental work. Data obtained from the RPT calibration should help in reconstructing the instantaneous positions of the tracer particle, which is in turn obtained from the tracking experiment. Therefore, for successful RPT measurement, the calculated number of counts via simulations or models for the RPT calibration process should reflect the actual RPT system. Thus, in the present contribution, we elected to apply the GEANT4-based Monte Carlo code to simulate the RPT calibration process for gas–liquid bubble column. In addition, the data obtained from the GEANT4 simulation will be compared against experimental data obtained using advanced automated RPT technique for the same investigated system at identical selected operating conditions as in a previous study [3].

## 2. The GEANT4 Model

The first step in using the GEANT4 is to simulate the investigated multiphase flow system (bubble column in this case) including the fraction and density of all materials. In this study, the RPT simulation was carried out for a gas–liquid bubble column made of plexiglass. The column has an inner dimeter of 5.5 in (0.14 m) and a height of 72 in (1.83 m). The column was designed without having any ports in the column’s wall to avoid any non-symmetric problems, which may complicate the simulation as well as the RPT reconstructions process. The liquid used is water, while the gas flow is air at an ambient temperature and pressure. In this study, only one of the operation conditions of the bubble column was selected to compare data obtained from the simulation with those from the actual RPT experimentation [3]. The selected operation condition is important to take into account the gas holdup distribution (void fraction) in the bubble column. However, for the selected operation conditions of the bubble column in this simulation, the averaged gas holdup distribution was already measured experimentally using gamma-ray computed tomography (CT) and previously reported by [3]. Hence, the averaged gas holdup distribution for the selected operation conditions is equal to 0.2 and this value will be taken to the simulation.

**Figure 1:**
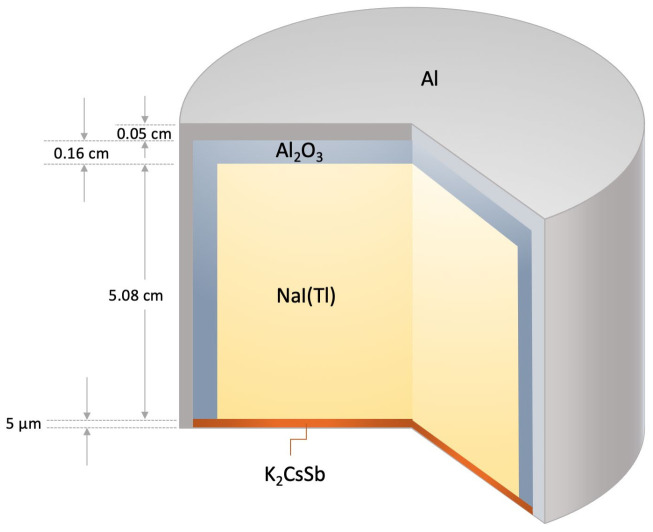
Geometrical of the sodium iodide NaI(Tl) detector (not to scale).

The next step in the simulation is to model the detectors’ type, configuration, and positions along the investigated system. In this simulation, 28 (5.08 cm × 5.08 cm) sodium iodide (NaI) detectors were used to track the gamma-rays emitted by the tracer. The detectors were arranged strategically around the bubble column at different axial levels in a spider manner. The optimal design of radioactive particle tracking experimentation found in the literature was highly considered in the detectors’ arrangement [11,33]. The detectors were positioned at 14 axial levels along the investigated system with respect to the expected bed dynamic of the bubble column. In each axial level, two detectors were positioned opposite to each (i.e., at a 180-degree angle to each other) to obtain efficient tracking of the tracer. In the actual RPT setup, the detectors were attached to movable horizontal aluminum structures which were consequently held by four vertical Unistrut bars equally distanced from the column. The four vertical Unistrut bars were divided at 90-degree intervals around the column, where each bar had seven detectors placed at the selected axial levels. Additionally, each detector was radially arranged at 12.7 cm with respect to the symmetrical axis of the column. The same detectors’ type, configuration, and positions of the actual RPT [3] were translated in the current GEANT4 simulation. Detectors’ specifications and dimensions were taken from the Canberra company through communication [34]. As shown in Figure 1, the simulated detector is 5.08 cm × 5.08 cm cylindrical NaI(Tl) crystal, covered with a 0.16 cm layer of Al_2_O_3_ reflector, and surrounded with a 0.05 cm thick Aluminum. A 5 µm photocathode layer of K_2_CsSb of the photomultiplier tube (PMT) was present at the end of the scintillation crystal instead of the whole PMT structure due to PMT complex shape. The materials densities of the NaI, Al_2_O_3_ reflector, aluminum, and K_2_CsSb photocathode were defined as 3.67g/cm^3^, 3.95 g/cm^3^, 2.7 g/cm^3^, and 2 g/cm^3^, respectively [34,35]. The scintillation emission spectrum of the NaI(Tl) crystal was included in the simulation to mimic the real optical properties of the crystal. The Scintillation light yield and decay time constant were taken as 38,000 photons/MeV and 250 nm, respectively. The refractive index was taken as 1.85 for the NaI(Tl) emission wavelength [35]. The GEANT4 electromagnetics standard physics library was implemented with optical physics option to transport photons and electrons. The GEANT4 code tracks interactions of each of the primary gamma events and their secondaries in the crystal. Based on the energy deposition within the NaI(Tl) crystal, optical photons were generated and tracked until they got absorbed by the photocathode. The total number of absorbed optical photons were recorded for each primary event. Once all of the primary events were completed, the pulse–height spectrum was built and calibrated into MeV energy unit.

**Figure 2:**
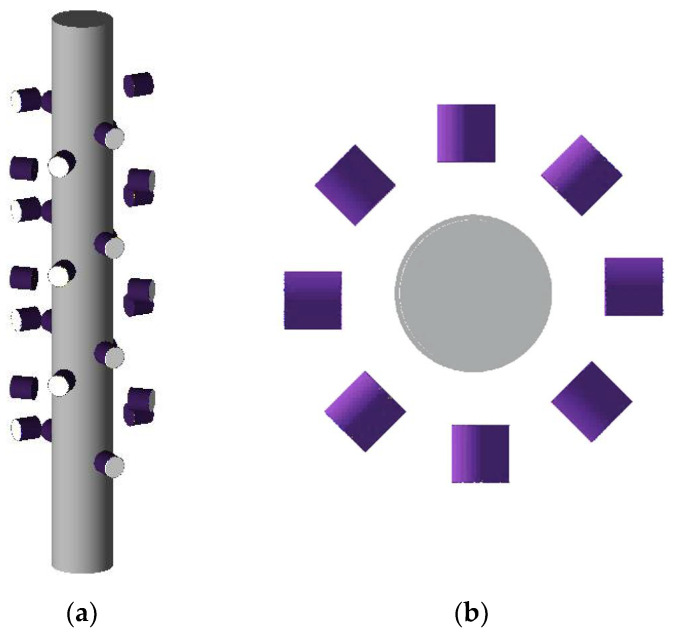
A snapshot of the GEANT4 simulation of the RPT detectors around the bubble column. (**a**) Angle view. (**b**) Top view.

The next step in the simulation is to model the tracer particle including the fraction and density of all materials. The radioactive source selected for the tracer preparation was Cobalt-60 (Co-60) (ds = 600 µm), with an initial activity of about 400 µci (microcurie). The Co-60 has 5.28-year halflife and has two photo peaks, one at 1173.2 keV and another at 1332.5 keV. The Co-60 (density = 8.9 g/cm^3^) was encapsulated with air in a polypropylene ball with a 2.3 mm outer diameter to obtain a composite tracer density similar to the water density. The same tracer particle of the RPT experimentation of [3] was defined in the current GEANT4 simulation. Figure 2 is a snapshot of the GEANT4 simulation showing the angle and top views of the 28 detectors surrounding the investigated bubble column. Detailed polar coordinates of the RPT detectors around the bubble column are given in Table 1.

**Figure 3:**
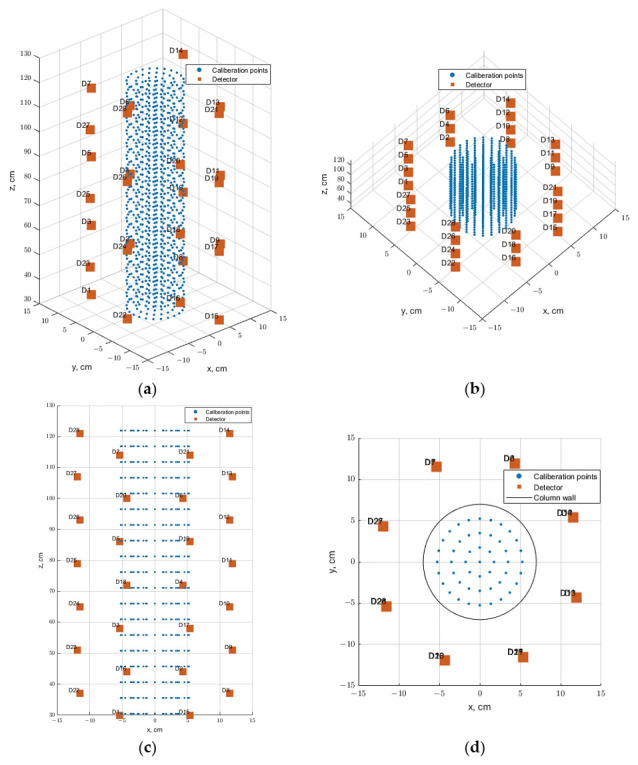
Simulated tracer positions and detectors configuration along the 5.5-inch bubble column: (**a**,**b**) 3D view, (**c**) Y-Z view, and (**d**) X-Y view or top view.

The last step is to define the tracer particle positions of the calibration process along the investigated multiphase flow system. In this work, total of 931 tracer particle positions along the 5.5-inch bubble column were conducted via the GEANT4. The tracer positions are distributed at 19 axial levels covering the bed dynamic of the bubble column where each level includes 49 positions. At each level, the 49 positions are distributed on three rings in the r-direction (0 cm, 1.75 cm, 3.5 cm, 5.25 cm) with different azimuthal positions. The simulated tracer positions as well as detector positions along the investigated bubble column are illustrated in Figure 3 in 3D/2D views.

## 3. Results and Discussion

### 3.1. The Response of a Single NaI(TI) Detector

**Figure 4:**
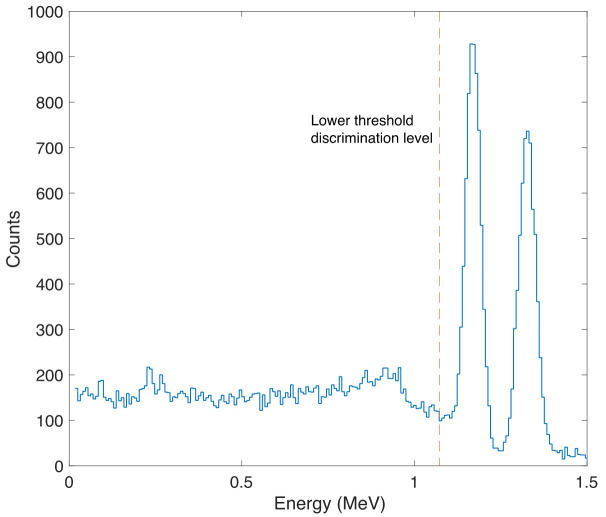
Pulse height spectrum of a simulated Co-60 radiation source collected in the simulated 5.08 cm × 5.08 cm) NaI detector showing the counting threshold employed in the GEANT4 model for all the detectors.

The pulse height spectrum of the simulated tracer particle (embedded with Co-60 radiation source) collected in the simulated (5.08 cm × 5.08 cm) NaI(Tl) detector is shown in Figure 4. The energy resolutions for simulated NaI(Tl) detector were determined to be 5.1% (FWHM) and 4.8% (FWHM) at 1.17 MeV and 1.33 MeV photo peaks, respectively. These values showed good agreement with typical NaI(Tl) detectors found in the literature [36,37]. Photons in the photo peaks are the original photons that travel without colliding from the source to the detector, and thus deposit all their energy within the NaI(Tl) detector. In the RPT experiment, counts recorded by the detector result from the number of photons incident on the detector, which is determined by the intrinsic efficiency of the detector. Of course, not all the photons incident on the detector are being counted, since some of the photons escape the detector crystal. Therefore, a threshold energy value that takes into account only the photons with energy within the photo peaks is usually set out in a real-life RPT system. This counting threshold was considered in our GEANT4 model and determined to be 1.075 MeV for all the 28 detectors.

### 3.2. Optimization of Optimal Primary Particles

**Figure 5:**
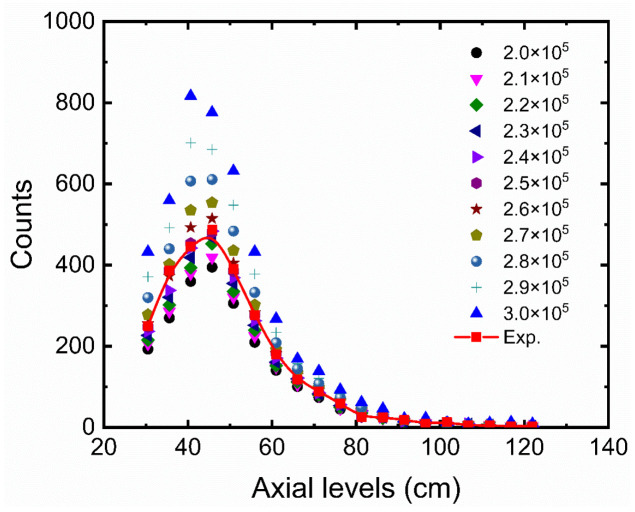
A comparison of counts obtained for different numbers of primary events adopted in the GEANT4 model with those obtained from RPT experiment.

**Figure 6:**
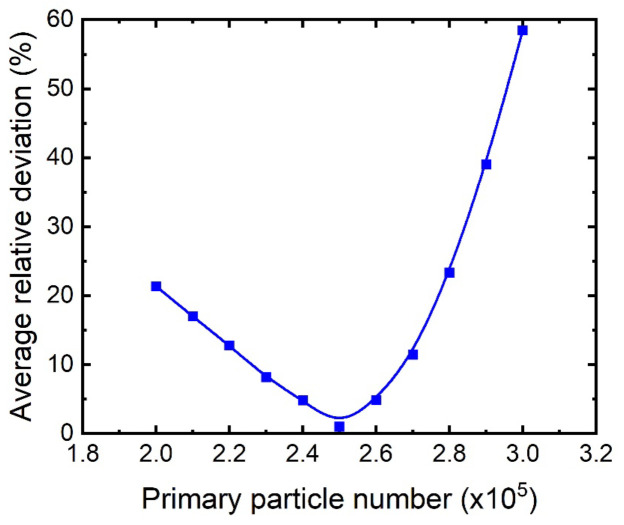
Absolute average relative deviation of the counts obtained for different numbers of primary events adopted from GEANT4 with comparison to the RPT experimental data.

As mentioned earlier, the object here is to simulate number of counts received by the detectors for the RPT calibration process. However, one should keep in mind that the produced simulation data are supposed to be used in the RPT reconstruction process to reconstruct the tracking data, which comes from an absolute experimental work. In other words, validation of the simulation results with some RPT experimental data of the calibration process is needed for these data to be used in the RPT reconstruction process. It is then required to match the RPT calibration process and data obtained throughout the GEANT4 model and error and trails. In the actual experiment, the radiation emitted by the radioactive particle was recorded by the detectors at a 0.02 s sampling time [3]. Taking into account the source activity (about 400 µCi), this means the source’s disintegration is equal to 400 × 10^−6^ × 3.7 × 10^10^ × 0.02 = 2.96 × 10^5^ disintegrations per second (dps) in each position. However, the RPT experimental detection system is subjected to the dead-time effect that reduces detection efficiency. Detector dead time is the minimum time required to record two successive particles interactions in the detection medium. Furthermore, dead time is the aggregate time of forming a pulse within the detection medium and the pulse processing time of the associated electronic [22]. Additionally, considering background radiation that would increases the number of the detectors’ interacted particles, which would lead to a rise in dead-time effect in the detection system. Consequently, optimizing numbers of primary particles (events) is required to be included in the GEANT4 simulation. Hence, the simulation was conducted for different numbers of primary events for 19 tracer positions along the axial levels at the axis of the bubble columns (i.e., the center). The evaluated number of primary events range from 200 K to 300 K with an increment of 10 K events. Figure 5 shows the comparison of counts obtained for different numbers of primary events with those obtained from the RPT experiment. For the convenience of comparison, the absolute average relative deviation (AARD) was calculated to assess the comparison between the counts obtained from the RPT experiment and the simulation for the evaluated primary events. Figure 6 showed that 250 K primary events had the lowest absolute average relative deviation (about 1%) with comparison to the RPT experimental data. Consequently, 2.5 × 10^5^ primary events were adopted for each tracer position in the GEANT4 simulation code.

### 3.3. Calibration Curves

**Figure 7:**
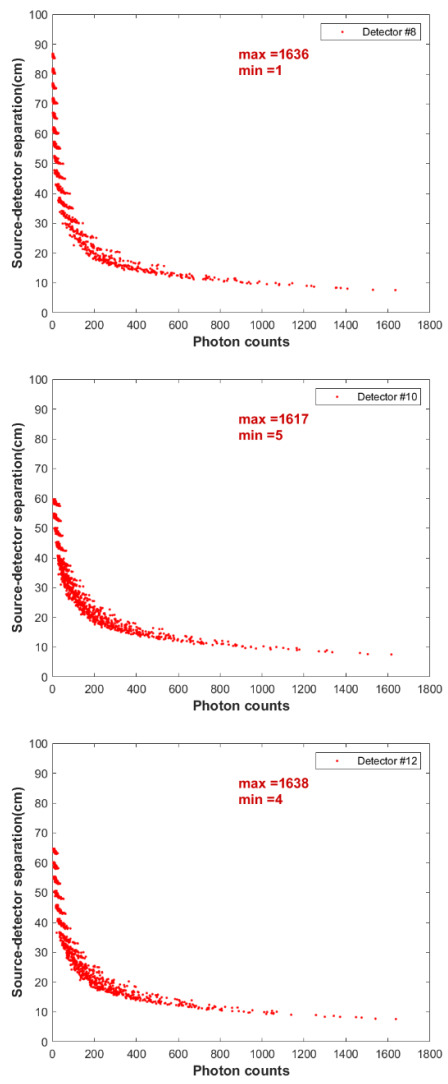

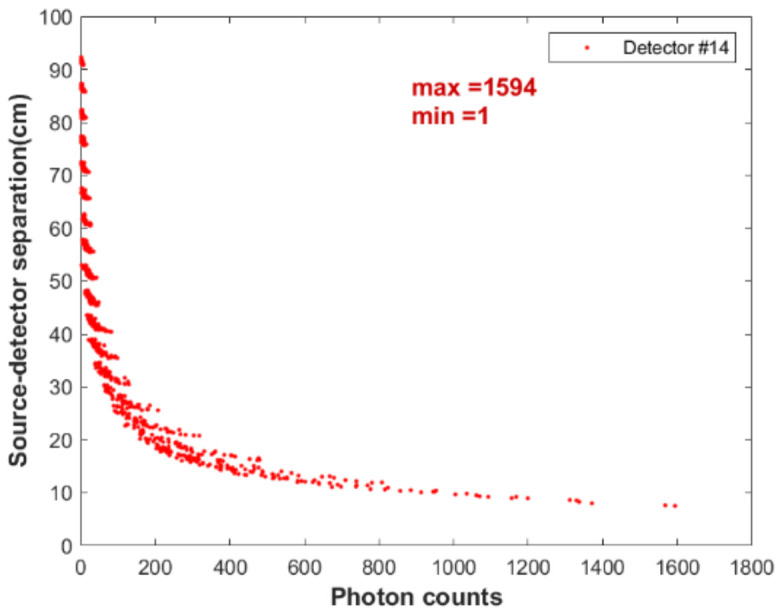
Distance count maps (calibration curve) predicted by the GEANT4 simulation for Detectors no. 8, 10, 12, and 14 for 931 tracer particle positions.

Following optimization of the simulation parameters of the GEANT4, the simulation was used to estimate the counts recorded by all the detectors for all the 931 known positions of the tracer particle. Figure 7 shows typical count versus distance obtained from the simulation for detectors no. 8, 10, 12, and 14 (along Bar#2). As shown in the figure, the recorded count by each detector increases as the source–detector separation distance decreases. The simulated photon counts recorded at the detector should represent the effective intensity of the tracer radiation. If no intervening medium is present, these recorded counts should follow an inverse-square relationship with distance. However, the distance count map is no longer an inverse-square relationship due to the difference in the attenuation of the photons caused by the intervening medium between the source and the detector. In addition, the trend of recorded counts exhibits a narrow decreases exponential curve, where data are not very scattered from each other, which favors the goodness of data obtained from either the RPT experiment or simulation. Additionally, it should be noted that the detector saturation effect is not detected for all the detectors owing to the prior preparation of the actual RPT setup with respect to detector positions. In the actual experiment, a case of detector saturation could occur when the rate of the photon reaching the crystal is higher than the photon counting rate of the detector.

### 3.4. The Response of Multiple Detectors as Function of the Tracer Position

**Figure 8:**
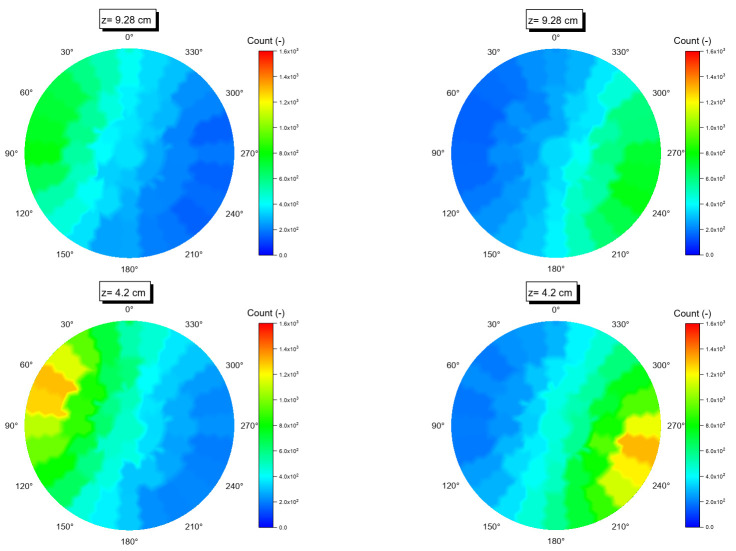

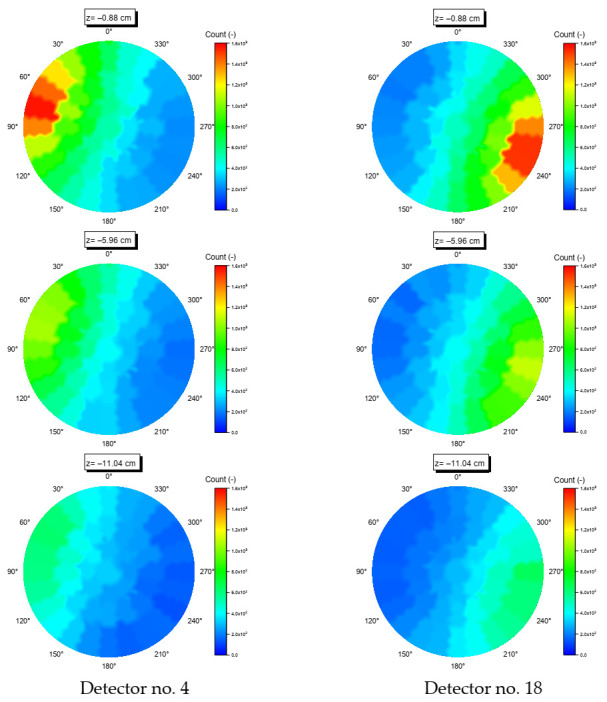
A typical distribution of simulated counts received by Detector no. 4 and Detector no. 18 as function of the axial distance from the detector axis.

**Figure 9:**
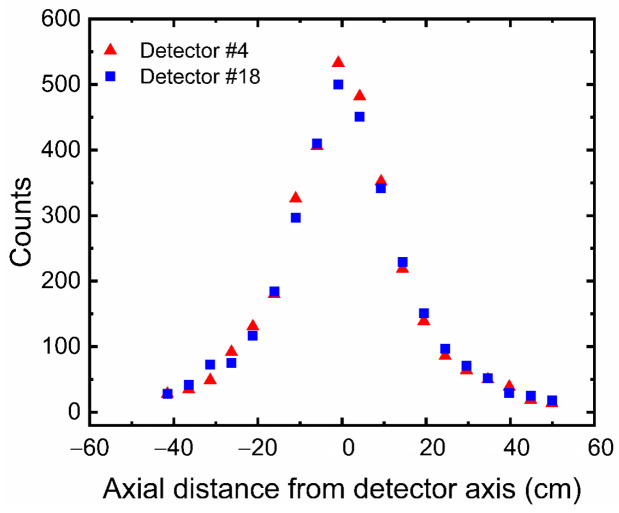
A comparison of simulated counts recorded by the two detectors when the tracer positions are in equal distance between the two detectors (i.e., along the axis of the column and perpendicular to the detector axis).

Figure 8 shows typical distribution of simulated counts received by Detector no. 4 and Detector no. 18 as function of axial distance from the detector axis. Taking into account that the two detectors are 180 degrees opposite of each other (Table 1), the count distributions are represented here to demonstrate the effective view of the two detectors. As expected, the maximum counts recorded by each detector take place when the tracer is closest to column wall and axially located near the axis of the detectors at z= −0.88. However, when the tracer positions are far from each detector, the recorded counts decrease. Additionally, it should be noted that effective view of each detector comes to a minimum end at a distance equal to 10 cm above and below each detector axis. Figure 9 shows a comparison of counts recorded by the two detectors when the tracer positions are in equal distance between the two detectors (i.e., along the axis of the column and perpendicular to detector axis). The maximum number of counts were found to be near the axis of the two detectors. However, when the tracer particle is moved along the axis of the column, the photon counts recorded at the simulation detectors die away rapidly due to the anisotropy effects of the solid angle. Such an effect was observed to occur for all simulated detectors.

### 3.5. Parity Plot (Simulation Versus Experimental)

**Figure 10:**
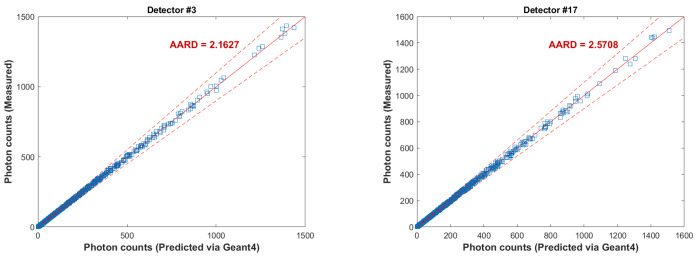

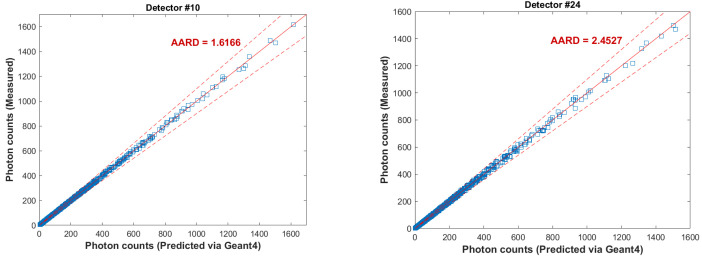
Parity plot of the predicted and measured counts different detectors shown the validity between the GEANT4 model employed in this study compared to the experimental data.

A parity plot of the calculated counts by the GEANT4 simulation and the measured counts is shown in Figure 10 for Detectors no. 3, 10, 17, and 24. The +5% and −5% limits are also shown in the figure. The simulated counts via the GEANT4 are in an excellent agreement with the measured counts via the experiment for all the detectors. The average absolute relative deviation (AARD) was calculated for all the detectors, where the maximum deviation was about 5% for Detector no. 28 and the minimum deviation was 1.5% for Detector no. 18. This demonstrates the validation of the GEANT4 model to estimate counts received by the detectors in the RPT calibration process.

## 4. Remarks

In the radioactive particle tracking (RPT) technique, a non-invasive measurement technique used to visualize multiphase flow systems, three process take place: calibration, tracking, and reconstruction. Successful RPT experimentation, and therefore, accuracy of data obtained from the technique, depend largely on the calibration process. Limitations in the RPT calibration process have been found in different experimental constraints and in assumptions made in the classical Monte Carlo approach used to simulate the number of counts received by the detectors. Therefore, in this study, an original GEANT4-based Monte Carlo simulation was developed to estimate the number of counts received by detectors in the RPT calibration process. The GEANT4 simulation was conducted for 28 detectors and 931 positions along the investigated multiphase flow system. The simulation results were satisfactory and showed a very good agreement with the experimental data, where the maximum deviation was about 5%. However, the results showed that the simulation over-predicted the counts when the number of primary events was not the optimal. This also emphasizes that optimization of modeling the RPT detection system is required. With the example of a gas–liquid bubble column, this study demonstrated that the use of the GEANT4-based Monte Carlo model was suitable to overcome limitations in the RPT calibration process. On the other hand, the GEANT4 simulation conducted in this study showed high computational cost. This, however, suggests that such a simulation could be coupled with empirical or semi models to produce large number of RPT calibration points at an optimum computational cost.

The GEANT4 version used in this study was 10.03. All 931 simulation positions were performed on a workstation Intl© Xeon© Gold 5115 CPU @ 2.40 GHz (20 CPUs), 64 GB RAM supported by Linux Ubuntu 18.04. The total computing time for the entire simulation was equal to 1200 h.

## Figures and Tables

**Table 1:** Polar coordinates of the RPT detectors around the bubble column used in the GEANT4 simulation.

Bar (#)	Detector (#)	r (cm)	θ (degree)	z (cm)	Bar (#)	Detector (#)	r (cm)	θ (degree)	z (cm)
**1**	1	12.7	115	30	3	15	12.7	295	30
	2		70	44		16		250	44
	3		115	58		17		295	58
	4		70	72		18		250	72
	5		115	86		19		295	86
	6		70	100		20		250	100
	7		115	114		21		295	114
**2**	8		25	37	4	22		205	37
	9		340	51		23		160	51
	10		25	65		24		205	65
	11		340	79		25		160	79
	12		25	93		26		205	93
	13		340	107		27		160	107
	14		25	121		28		205	121

## Data Availability

Not applicable.

## Abbreviations

**Symbols**		**Description**
C	:	Number of photopeak photon counts recorded by a detector.
T	:	Time interval during which C is recorded.
υ	:	Number of γ-ray photons emitted per disintegration.
A	:	Strength of the radioactive source.
ϕ	:	The probability that a γ-ray will emerge from the reactor without scattering and will interact with the detector.
ε	:	The absolute detection efficiency.
τ	:	The dead time of the counting system per accepted pulse.
Ω	:	The solid angle subtended by the detector.
r	:	Separation distance between the source and the point on the surface of the detector.
$\underline{n}$	:	The radius vector from the source to the detector.
$f_{a}$	:	Probability that gamma-rays traveling within solid angle Ω will not interact with the medium inside the reactor and the wall of reactor.
$f_{D}$	:	Probability that gamma-rays traveling distance d will interact in the crystal material.
$\mu_{R}$	:	The mass attenuation coefficient of the reactor inventory (for the energy of the γ-ray photon).
$e_{R}$	:	The penetration depth through the medium inside the reactor.
$\mu_{w}$	:	The mass attenuation coefficient of the reactor wall (for the energy of the γ-ray photon).
$e_{w}$	:	The penetration depth through the medium inside the reactor wall.
$\mu_{D}$	:	The mass attenuation coefficient of the detector crystal material (for the energy of the γ-ray photon).
d	:	The penetration depth through the medium inside the thickness of crystal inside the detector
dΣ	:	Infinitesimal surface area element of the detector.
